# Prognostic value of metabolic parameters on preoperative 18F-Fluorodeoxyglucose positron emission tomography/computed tomography in patients with stage III gastric cancer

**DOI:** 10.18632/oncotarget.11574

**Published:** 2016-08-24

**Authors:** Sae Jung Na, Joo Hyun O, Jae Myung Park, Han Hee Lee, Sung Hak Lee, Kyo Young Song, Myung-Gyu Choi, Cho Hyun Park

**Affiliations:** ^1^ Department of Radiology, The Catholic University of Korea, Uijeongbu St. Mary's Hospital, Seoul, Korea; ^2^ Department of Radiology, The Catholic University of Korea, Seoul St. Mary's Hospital, Seoul, Korea; ^3^ Department of Internal Medicine, The Catholic University of Korea, Seoul St. Mary's Hospital, Seoul, Korea; ^4^ Department of Hospital Pathology, The Catholic University of Korea, Seoul St. Mary's Hospital, Seoul, Korea; ^5^ Department of Surgery, The Catholic University of Korea, Seoul St. Mary's Hospital, Seoul, Korea

**Keywords:** positron emission tomography, gastric cancer, prognosis, tumor metabolism

## Abstract

This study investigated the prognostic value of metabolic parameters determined by 18F-fluorodeoxyglucose (FDG) positron emission tomography/computed tomography (PET/CT) in patients with stage III gastric cancer. Patients with pre-operative PET/CT and confirmed stage III after curative surgical resection were retrospectively enrolled. Parameters evaluated from pre-operative PET/CTwere maximum standardized uptake value (SUV_max_) and peak SUV (SUV_peak_) of primary tumor, SUV_max_ or SUV_peak_ of tumor to liver ratio (TLR_max_ and TLR_peak_). Volumetric parameters, metabolic tumor volume (MTV) and total lesion glycolysis (TLG), were also evaluated. These PET/CT parameters were compared with the overall survival (OS) and recurrence-free survival (RFS). From total of 133 consecutive patients, tumor recurrence was found in 54 patients (40.6%) and 53 died during the follow-up period (median, 43 mo; range 5-62). In univariate analysis, SUV_max_, SUV_peak_, TLR_max_ and TLR_peak_ were significantly associated with the OS and RFS. In multivariate analysis, high TLR_max_ and TLR_peak_ were significantly unfavorable prognostic factors for RFS (both *P*<0.05) even after adjusting for age, depth of tumor invasion, lymph node metastasis, and chemotherapy. MTV and TLG showed no statistically significant correlation with outcome. In conclusion, glucose metabolism of primary tumor measured by pre-operative PET/CT provides prognostic information, especially for recurrence, in stage III gastric cancer.

## INTRODUCTION

18F-fluorodeoxyglucose (FDG) has been used in the detection of variable cancers since the advance of positron emission tomography/computed tomography (PET/CT) hybrid imaging system. Among the 18F-FDG PET/CT parameters, the highest SUV from a single pixel anywhere within the tumor, the maximum standardized uptake value (SUV_max_), is the most commonly documented parameter in cancer studies. Recently, the highest mean SUV from small fixed dimension, 1 cm^3^ spherical volume of interest centered over the highest uptake part of the tumor, so called peak standardized uptake value (SUV_peak_) was proposed as a more robust measurement [1, 2]. PET parameters are alternatively presented as ratios to the normal background activity, such as liver or blood pool, because the SUV ratio is less influenced by noise and image resolution, provides internal normalization, and therefore more suitable for studies using different scanners [3].

Some studies showed that glucose uptake of primary tumor mass by 18F-FDG PET/CT evaluation correlated with the prognosis of patients in a number of different cancer types [4–6]. In gastric cancer, preoperative 18F-FDG PET/CT evaluation has demonstrated limited clinical role because of its variable sensitivity for primary tumor and lymph node metastasis, of which sensitivity as low as 26% and 23%, respectively, have been reported [7–11]. The accuracy of PET/CT parameters depended on the tumor stage and histopathologic features [12].

Recent studies showed that gastric cancers have high prevalence of the genetic mutations which are related with metabolic changes [13] and that overexpression of the metabolism related signaling proteins were correlated with poor prognostic factors [14]. Such findings of altered metabolism in gastric cancer suggest that the parameters represented by 18F-FDG PET/CT images could be related with the biologic characteristics of gastric cancers, and the patient's clinical prognosis.

Several studies have evaluated the role of 18F-FDG PET/CT in predicting clinical outcome of patients with gastric cancers [9, 10, 15–18]. These studies included a small number of patients, had a substantial portion of stage I cancers which did not allow for accurate measurement of the metabolic FDG PET parameters, evaluated patients with metastatic cancers in whom tumor recurrence could not be gauged, or measured only one or two simple metabolic FDG PET parameters. Furthermore, the results among these previous studies were contradictory.

We evaluated the prognostic value of multiple metabolic FDG PET parameters from preoperative 18F-FDG PET/CT in patients with curative surgical resection of stage III gastric cancer.

## RESULTS

### Patient characteristics

A total of 133 consecutive patients (86 males, 47 females) were included. Their mean age was 60.1 ± 12.0 years, and median follow up time was 43 months (range, 5-62 months). In most of the patients (84%, 112/133) in this study, D2 lymphadenectomy was performed. Fifty four patients (40.6%) experienced recurrence and 53 (39.8%) died during the follow up period. The median follow-up period was 42 months (range 0-62 months). The patient characteristics are summarized in Table 1.

**Table 1:** Patient Demographics (N=133)

Characteristics	Number (%)
Age (years)	
≤ 65	83 (62.4%)
> 65	50 (37.6%)
Sex	
Male	86 (64.7%)
Female	47 (35.3%)
Depth of tumor invasion	
T2	4 (3.0%)
T3	33 (24.8%)
T4a	92 (69.2%)
T4b	4 (3.0%)
Lymph node metastasis	
N1	18 (13.5%)
N2	50 (37.6%)
N3a	35 (26.3%)
N3b	30 (22.6%)
Pathologic stage	
IIIa	41 (30.8%)
IIIb	44 (33.1%)
IIIc	48 (36.1%)
Lauren classification	
Intestinal	37 (27.8%)
Non-intestinal	96 (72.2%)
Lymphatic invasion	
Yes	130 (97.7)
No	3 (2.3)
Vein invasion	
Yes	112 (84.2)
No	21 (15.8)
Histopathologic type	
Differentiated	45 (33.8%)
Undifferentiated	88 (66.2%)
Surgery	
Subtotal gastrectomy	75 (56.4%)
Total gastrectomy	58 (43.6%)
Lymph node dissection	
D1	21 (15.8%)
D2	112 (84.2%)
Adjuvant chemotherapy	
No	10 (7.5%)
Yes	123 (92.5%)

### Clinicopathologic characteristics and metabolic parameters

In all patients, the median values of SUV_max_, SUV_peak_, SUV_max_ of tumor to SUV_mean_ of liver ratio (TLR_max_) and SUV_peak_ of tumor to SUV_mean_ of liver ratio (TLR_peak_) were 5.5 (range, 1.8 - 24.3), 4.4 (range, 1.5 - 19.2), 2.8 (range, 1.0 - 15.2), and 2.3 (range, 0.8 - 10.6), respectively. Stage IIIb and IIIc tumors showed significantly higher values for all of the FDG PET parameters (Table 2). The intestinal and diffuse type tumors also showed significantly higher SUV_max_, SUV_peak_, TLR_max_, and TLR_peak_ than the mixed type tumors. Low grade tumors showed significantly higher levels for all these 18F-FDG PET metabolic parameters than the high grade tumors (Table 2). The tumors with venous invasion also showed significantly higher values of these 18F-FDG PET parameters. However, there were no statistical differences for each of the SUV_max_, SUV_peak_, TLR_max_ and TLR_peak_ parameter when analyzed according to age, sex, the depth of tumor invasion, extent of lymph node metastasis, the lymphatic invasion of tumor cells, and extent of lymph node dissection (D1 or D2). In the primary tumors, visually positive FDG uptake was noted in 100 patients (75.2%). Among them, the median of metabolic tumor volume (MTV; the tumor volume computed from pixels showing higher SUV than the designated threshold) and total lesion glycolysis (TLG; the product of MTV and the mean SUV of the tumor lesion) were 17.2 cm^3^ (range 0 – 231.2 cm^3^) and 73.8 g·cm^3^/ml (range 0 – 1127.9), respectively. The MTV was higher in patients with vein invasion than without vein invasion (p=0.048). MTV and TLG showed no significant correlation with the clinicopathologic parameters otherwise.

**Table 2:** Clinicopathologic characteristics and metabolic parameters

			SUV_max_		SUV_peak_		TLR_max_		TLR_peak_	
		N	Median (range)	*P*	Median (range)	*P*	Median (range)	*P*	Median (range)	*p*
Total		133	5.5 (1.8 - 24.3)		4.4 (1.5 - 19.2)		2.8 (1.0 - 15.2)		2.3 (0.8 - 10.6)	
Age (years)	≤ 65	83	4.7 (1.8-22.8)	0.170	4 (1.5-17.8)	0.206	2.6 (1-12.5)	0.099	2.1 (0.8-9)	0.107
	> 65	50	5.95 (1.8-24.3)		4.8 (1.5-19.2)		3.25 (1.1-15.2)		2.6 (0.9-10.6)	
Sex	Male	86	6 (1.8-22.8)	0.286	4.95 (1.5-17.8)	0.225	3.2 (1-12.5)	0.279	2.55 (0.8-9)	0.256
	Female	47	4.7 (2.2-24.3)		3.7 (1.9-19.2)		2.4 (1-15.2)		2 (0.8-10.6)	
Depth of tumor invasion	T2	4	3.9 (2.1-6.1)	0.404	3.25 (1.8-4.9)	0.393	2.45 (1.4-3.6)	0.592	2.05 (1.2-2.9)	0.549
	T3	33	4.5 (1.8-24.3)		3.6 (1.5-16.9)		2.5 (1-15.2)		2.1 (0.8-10.6)	
	T4a	92	5.95 (2.2-23.7)		4.8 (2-19.2)		3.15 (1.2-11.9)		2.5 (1-9.6)	
	T4b	4	7.45 (3.2-12.7)		5.55 (2.9-10.4)		4.35 (1.8-6.4)		3.25 (1.6-5.2)	
Lymph node metastasis	N1	18	3.8 (2.6 - 22.8)	0.123	3.3 (2.3-17.8)	0.142	2.15 (1.2-9.9)	0.168	1.8 (1-7.7)	0.170
	N2	50	4.65 (1.8 - 24.3)		3.75 (1.5-19.2)		2.8 (1-15.2)		2.15 (0.8-10.6)	
	N3a	35	5.5 (2.1 - 18.3)		4.6 (1.7-15.3)		3.1 (1.4-10.1)		2.3 (1-7.7)	
	N3b	30	6.1 (1.8 - 20.1)		5.25 (1.5-15.8)		3.4 (1-9.1)		2.85 (0.8-7.2)	
Pathologic stage	IIIa	41	4.3 (1.8-24.3)	0.009	3.4 (1.5-17.8)	0.011	2.3 (1-15.2)	0.023	2 (0.8-10.6)	0.032
	IIIb	44	5.85 (1.8-23.7)		4.5 (1.5-19.2)		3.25 (1-11.9)		2.5 (0.8-9.6)	
	IIIc	48	6.3 (2.5-20.1)		5.3 (2.2-15.8)		3.4 (1.2-9.2)		2.7 (1-7.7)	
Lymphatic invasion	Absence	3	4.5 (2.6-7)	0.449	3.5 (2.3-5.7)	0.426	2.6 (1.3-4.1)	0.565	2.1 (1.2-3.4)	0.565
	Presence	130	5.5 (1.8-24.3)		4.4 (1.5-19.2)		2.8 (1-15.2)		2.3 (0.8-10.6)	
Venous invasion	Absence	112	4.65 (1.8-24.3)	0.004	3.75 (1.5-19.2)	0.003	2.6 (1-15.2)	0.003	2.1 (0.8-10.6)	0.002
	Presence	21	7 (3.2-22.8)		5.7 (2.9-17.8)		4.1 (1.8-9.9)		3.4 (1.6-7.7)	
Lauren classification	Intestinal	37	7 (2.4-24.3)	< 0.001	5.3 (2.2-17.8)	< 0.001	3.6 (1.4-15.2)	0.001	2.9 (1.3-10.6)	0.001
	Diffuse	55	6.1 (1.8-23.7)		5.1 (1.5-19.2)		3.2 (1-12.5)		2.6 (0.8-9.6)	
	Mixed	41	3.9 (2.1-16.7)		3.3 (1.7-13.1)		2.1 (1-9.3)		1.8 (0.8-7.3)	
Grade	Low	45	7 (2.4-24.3)	0.012	5.3 (2.2-17.8)	0.021	3.5 (1.4-15.2)	0.019	2.8 (1.2-10.6)	0.036
	High	88	4.8 (1.8-23.7)		4.05 (1.5-19.2)		2.5 (1-12.5)		2.1 (0.8-9.6)	

SUV_max_, maximum standardized uptake value; SUV_peak_, peak standardized uptake value; TLR_max_, SUV_max_ of tumor to SUV_mean_ of normal liver ratio; TLR_peak_, SUV_peak_ of tumor to liver ratio

### Prognostic value of PET for survival

**Figure 1:**
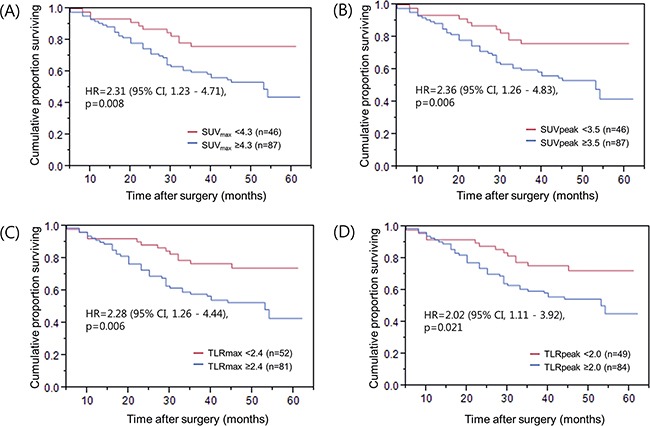
Overall survival of patients grouped by 18F-FDG PET parameters **A.** SUV_max_, **B.** SUV_peak_, **C.** TLR_max_, and **D.** TLR_peak_ values

The impact of 18F-FDG PET metabolic parameters on clinical outcome, as measured by patient overall survival (OS) and recurrence free survival (RFS), was calculated. The optimal cutoff values for SUV_max_, SUV_peak_, TLR_max_, TLR_peak_ were 4.3, 3.4, 2.4 and 2.0, respectively. Patient age, lymph node metastasis and TNM stages were significantly associated with OS in stage III gastric cancer patients (Table 3). As shown in Figure 1A to 1D, the Kaplan-Meier curves showed survival difference between the tumors with high and low values of SUV_max_, SUV_peak_, TLR_max_ and TLR_peak_, in which the patients with high 18F-FDG PET parameter values had shorter OS (Table 3). However, these metabolic parameters were not prognostic after adjusting for other clinical factors including patient age, depth of tumor invasion, lymph node metastasis, and adjuvant chemotherapy (Table 3).

**Table 3:** Univariate and multivariate analysis for overall survival

Characteristics	n (%)	Univariate analysis			Multivariate analysis*		
		HR	95% CI	*P*	HR	95% CI	*P*
Age (years)							
≤ 65	83 (62.4%)	1					
> 65	50 (37.6%)	1.78	1.038 - 3.06	0.04			
Sex							
Female	86 (64.7%)	1					
Male	47 (35.3%)	1.21	0.69 - 2.21	0.51			
Depth of invasion							
T2/T3	37 (27.8%)	1					
T4a/b	96 (72.2%)	1.78	0.93 - 3.76	0.08			
Lymph node metastasis							
N1/N2	68 (51.1%)	1					
N3a/b	65 (48.9%)	3.21	1.82 - 5.94	<.0001			
Pathologic stage							
IIIa	41 (30.8%)	1					
IIIb	44 (33.1%)	1.74	0.76 - 4.19	0.19			
IIIc	48 (36.1%)	4.00	1.97 - 8.95	<.0001			
Lauren classification							
Intestinal	37 (27.8%)	1					
Non-intestinal	96 (72.2%)	0.93	0.52 - 1.71	0.80			
Histopathologic grade							
Low	45 (33.8%)	1					
High	88 (66.2%)	0.99	0.57 - 1.78	0.98			
Surgery							
Subtotal gastrectomy	75 (56.4%)	1					
Total gastrectomy	58 (43.6%)	0.99	0.57 - 1.71	0.98			
Adjuvant chemotherapy							
No	10 (7.5%)	1					
Yes	123 (92.5%)	0.55	0.256 - 1.44	0.21			
18F-FDG PET parameters							
Visualization							
negative	33 (24.8%)	1			1		
positive	100 (75.2%)	1.25	0.67 - 2.56	0.49	0.89	0.45 - 1.89	0.76
SUV_max_							
< 4.3	46 (34.6%)	1			1		
≥ 4.3	87 (65.4%)	2.31	1.23 - 4.71	0.008	1.47	0.74 - 3.11	0.28
SUV_peak_							
<3.5	46 (34.6%)	1			1		
≥3.5	87 (65.4%)	2.36	1.26 - 4.83	0.006	1.50	0.76 - 3.20	0.25
TLR_max_							
<2.4	52 (39.1%)	1			1		
≥2.4	81 (60.9%)	2.28	1.26 - 4.44	0.006	1.55	0.82 - 3.10	0.18
TLR_peak_							
<2.0	49 (36.8%)	1			1		
≥2.0	84 (63.2%)	2.02	1.11 - 3.92	0.021	1.51	0.81 - 2.98	0.20

CI, confidence interval; PET, positron emission tomography; SUV_max_, maximum standardized uptake value; SUV_peak_, peak standardized uptake value; TLR_max_, SUV_max_ of tumor to SUV_mean_ of normal liver ratio; TLR_peak_, SUV_peak_ of tumor to liver ratio; HR, hazard ratio

* adjusted for age, depth of tumor invasion, lymph node metastasis, and chemotherapy

**Figure 2:**
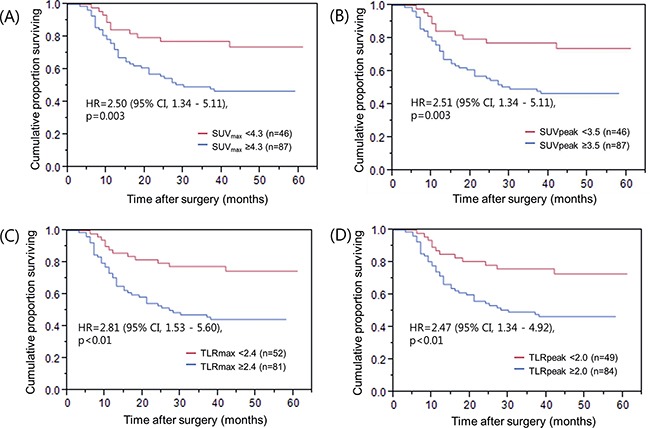
Recurrence-free survival of patients grouped by 18F-FDG PET parameters **A.** SUV_max_ values, **B.** SUV_peak_, **C.** TLR_amx_, and **D.** TLR_peak_ values

Kaplan-Meier analysis showed that clinical factors including depth of tumor invasion, lymph node metastasis and TNM stage was significantly associated with RFS (Figure 2A to 2D). Among the metabolic parameters, higher SUV_max_, SUV_peak_, TLR_max_, and TLR_peak_ was significantly associated with shorter RFS, even after adjusting for other clinical factors including patient age, depth of tumor invasion, lymph node metastasis, and adjuvant chemotherapy (Table 4).

**Table 4:** Univariate and multivariate analysis for recurrence-free survival

Characteristics	n (%)	Univariate analysis			Multivariate analysis*		
		HR	95% CI	*P*	HR	95% CI	*P*
Age (years)							
≤ 65	83 (62.4%)	1					
> 65	50 (37.6%)	1.46	0.84 - 2.50	0.17			
Sex							
Female	86 (64.7%)	1					
Male	47 (35.3%)	1.04	0.60 - 1.86	0.88			
Depth of invasion							
T2/T3	37 (27.8%)	1					
T4a/b	96 (72.2%)	2.45	1.22 - 5.62	0.01			
Lymph node metastasis							
N1/N2	68 (51.1%)	1					
N3a/b	65 (48.9%)	2.25	1.30 - 3.98	0.003			
Pathologic stage							
IIIa	41 (30.8%)	1					
IIIb	44 (33.1%)	2.92	1.26 - 7.55	0.012			
IIIc	48 (36.1%)	5.22	2.42 - 12.95	<0.001			
Lauren classification							
Intestinal	37 (27.8%)	1					
Non-intestinal	96 (72.2%)	0.62	0.36 - 1.11	0.10			
Histopathologic grade							
Low	45 (33.8%)	1					
High	88 (66.2%)	0.75	0.44 - 1.30	0.30			
Surgery							
Subtotal gastrectomy	75 (56.4%)	1					
Total gastrectomy	58 (43.6%)	0.82	0.47 - 1.41	0.47			
Adjuvant chemotherapy							
No	10 (7.5%)	1					
Yes	123 (92.5%)	0.86	0.38 - 2.48	0.75			
18-FDG PET parameters							
Visualization							
negative	33 (24.8%)	1			1		
positive	100 (75.2%)	1.82	0.94 - 3.99	0.08	1.39	0.69 - 3.10	0.38
SUV_max_							
< 4.3	46 (34.6%)	1			1		
≥ 4.3	87 (65.4%)	2.50	1.34 - 5.11	0.003	1.89	0.97 - 3.97	0.06
SUV_peak_							
<3.5	46 (34.6%)	1			1		
≥3.5	87 (65.4%)	2.51	1.34 - 5.11	0.003	1.84	0.94 - 3.86	0.08
TLR_max_							
<2.4	52 (39.1%)	1			1		
≥2.4	81 (60.9%)	2.81	1.53 - 5.60	<0.001	2.24	1.18 - 4.57	0.01
TLR_peak_							
<2.0	49 (36.8%)	1			1		
≥2.0	84 (63.2%)	2.47	1.34 - 4.92	0.003	2.07	1.10 - 4.17	0.02

SUV_max_, maximum standardized uptake value; SUV_peak_, peak standardized uptake value; TLR_max_, SUV_max_ of tumor to SUV_mean_ of normal liver ratio; TLR_peak_, SUV_peak_ of tumor to liver ratio; HR, hazard ratio

* adjusted for age, depth of tumor invasion, lymph node metastasis, and adjuvant chemotherapy

The optimal cutoff values for prognosis analysis of MTV and TLG were 4.2 cm^3^ and 14.3 cm^3^, respectively. In Kaplan-Meier analysis of volumetric parameters, higher TLG tended to show poorer prognosis for OS (both p=0.072). However, recurrence free survival was not associated with these volumetric parameters.

## DISCUSSION

This study investigated the prognostic value of PET parameters measured from pre-resection 18F-FDG PET/CT in patients with gastric cancer. We found that metabolic 18F-FDG PET parameters were associated with tumor stage, lymphovascular invasion, Lauren's classification, and tumor grade in these patients with stage III gastric cancer. We also observed that higher metabolic PET parameter values were associated with worse overall prognosis for the patients. Furthermore, metabolic parameters from 18F-FDG PET/CT could predict tumor recurrence in this population of stage III gastric cancers, which was independent of other well-known clinical prognostic factors such as patient age, depth of tumor invasion, and lymph node metastasis.

Several previous studies evaluated the prognostic role of metabolic activity of primary gastric tumor measured from 18F-FDG PET/CT [9, 15–17, 19–21]. Except studies which evaluated metastatic gastric cancers [16, 21], most enrolled patients of all tumor stages, from early to advanced [15, 17, 19]. The FDG avidity could be underestimated when gastric cancer is in the early stages because of partial volume effect resulting from the small sized tumor [8, 10]. Therefore, if many early stage patients are involved in a study, it can exaggerate the ability of PET to predict prognosis. In order to avoid these concerns and focus on the correlation between prognosis and tumor metabolism itself, we included consecutive patients with only stage III gastric cancer.

18F-FDG PET/CT has limitations in the evaluation of gastric cancer because of relatively frequent false negativity [9]. Prior studies showed that the SUV was positively associated with tumor stages [10, 22]. Our study showed that pathologic tumor characteristics were also associated with the glucose metabolism of gastric tumor. Consistent with a few previous studies [9, 11], we also found that the intestinal type by Lauren classification and low grade malignant group showed significantly higher FDG uptake compared to the non-intestinal type and high grade group. Although histologic grades have been known conventionally to be related with biologic behavior, it is still a controversial finding [23] [24]. Our study showed no significantly different prognosis between low and high grade malignant groups.

The alerted metabolic pathways in tumor cells can be from direct response to growth factor signaling, and results from active reprogramming by altered oncogenes and tumor suppressors [25, 26]. The PI3K/AkT/mTOR pathway activated by growth factors enhances glucose uptake and glycolysis, and also tumorigenesis directly by reprogramming the mitochondria [27, 28]. Especially in gastric cancer, high prevalence of the mutation of related gene such as PI3KA has been reported [13], and a recent study showed overexpression of the majority of the proteins involved in PI3K/AkT/mTOR pathway and their correlation with pathologic factors of poor prognosis [14]. It is well known that the FDG uptake is associated with the expression of glucose transporter-1 (GLUT-1) [29] and a study presented that GLUT-1 was a potent candidate for predicting prognosis in patient with gastric cancer [30]. These findings support that the metabolic activity of tumor measured by FDG PET could reveal the aggressiveness of tumor.

Although the most aggressive focus within a tumor may be the most important in explaining the biologic behavior of the entire tumor when viewed from the theory of cancer stem cell, total tumor volume and its metabolic activity have also been of interest and importance when characterizing a tumor [1]. Therefore, there have been many studies using volumetric parameters such as MTV or TLG measured by PET/CT in various cancers [31–33]. We evaluated these volumetric parameters from 100 patients who showed visually perceptible FDG uptake in tumor, but they showed no statistical significance for predicting prognosis in our cohort. Normal physiologic gastric activity, underlying inflammation, and wide range of metabolic activity of gastric cancer could be hurdles for accurate quantification, especially compare to other cancers. For exact measurement, we referred to endoscopy and enhanced CT that were performed as a routine staging work up. Furthermore, we could reduce the error in measuring PET parameters, because patients in this study had stage III gastric cancer. However, measuring volumetric parameters of gastric cancer is actually not easy. To reduce the error of tumor delineation, we measured the volumetric parameters only when the tumor showed perceptible 18F-FDG activity. Also, empirical threshold of tumor delineation was quite high, 3.0, compared to the values reported for other cancer type mostly in the range of 2 to 2.5. Further studies about reader variability of volumetric parameters would be needed in gastric cancer.

In gastric cancer, tumor progression and aggressiveness are represented by tumor size, stage and the status of lymph node metastasis, which are well-known and widely used prognostic factors [12]. However, such factors cannot be evaluated accurately prior to invasive surgery. Furthermore, even in stage III gastric cancers, variable prognoses are presented even with post-operative adjuvant chemotherapy. Therefore, many studies to evaluate prognostic biomarkers have reported [34, 35] and clinical trials are currently underway about optimal postoperative treatments in these patients (ClinicalTrials.gov, NCT01618474, NCT01935778). Besides the undetermined optimal use of these agents, there has been an unmet need about defining the patients who did not benefit with adjuvant chemotherapy. Our results showed that PET/CT parameters were positively associated with pathologic stage IIIA-C. Furthermore, TLRmax and TLRpeak were independent factors for predicting recurrence after adjusting for T and N stages. In this study cohort, there is no different proportion of patient with D2 lymph node dissection between high and low metabolic groups. These imply that metabolic status of gastric cancer is an independent prognostic factor and that PET parameters can be used as an imaging biomarker. Even though therapeutic strategy cannot be applied directly with the current data, more active surveillance program can be applied to the patients with gastric cancers demonstrating high metabolic activity on 18F-FDG PET/CT.

Our study has some limitations. First, the sample size was not based on the power calculation. However, this was not able to be easy because the previous studies did not arrived at the same results and the cutoff-values from 18F-FDG PET parameters were different among these studies. Second, present study was analyzed in a stage. Therefore, our results have limitation to generalize to the other stages. Third, we used different two PET/CT scanners. Even though we could not do cross calibration between two scanners at the time of the imaging. So, we also measured the 18F-FDG uptake target to liver ratio normalized to the internal reference organ of the liver to reduce the problems related to different scanners.

In conclusion, metabolic activity of primary gastric tumor quantitatively computed from 18F-FDG PET/CT is a prognostic factor in patients with stage III gastric cancer. In particular, 18F-FDG PET/CT may guide optimized management plan and decision making process in the subset of stage III gastric cancer patients who are surgically treated. For this purpose, further prospective studies should be performed to establish the role of 18F-FDG PET/CT in these patients.

## MATERIALS AND METHODS

### Patients

We retrospectively enrolled patients from January 2009 to December 2010 at Seoul St. Mary's hospital, who confirmed to have stage III gastric cancer after curative surgical resection. All patients with gastric cancer underwent FDG PET/CT prior to therapy. Clinicopathologic data were retrospectively reviewed. We excluded the patients who expired due to surgical complications and who underwent neoadjuvant chemotherapy before 18F-FDG PET/CT evaluation. All enrolled patient underwent total or subtotal gastrectomy with D1 or D2 lymph node dissection. Tumor staging was done based on the TNM classification proposed by the Union International Cancer Control 7th edition [36]. This study was approved by the Institutional Review Board of Seoul St. Mary's Hospital (KC14RISI0834) and the requirement to obtain informed consent was waived.

### Histopathologic variables

We assessed the relationships between the following histopathologic variables: depth of tumor invasion, nodal metastasis, and histologic grade of differentiation. According to the Lauren classifications, the gastric tumors were categorized into intestinal and diffuse types. The presence of tumor cell invasion within venous or lymphatic channels was noted as well. Patients with lymph node metastasis received adjuvant chemotherapy after the operation, except in cases with patient refusal or poor medical conditions (comorbidity) such as liver cirrhosis or renal failure. For the grade of differentiation, the histopathologic type at the primary site was categorized as papillary adenocarcinoma, well differentiated adenocarcinoma, moderately differentiated adenocarcinoma, poorly differentiated adenocarcinoma, and signet-ring cell carcinoma according to the World Health Organization classification with Japanese modification [37, 38]. For statistical analysis, we identified the first 3 types of differentiation as a ‘low-grade malignancy group’ and the latter 2 types as a ‘high-grade malignancy group’ according to the conventionally accepted relationship between the types of cancer and biologic behavior.

### 18F-FDG PET/CT protocol and image analysis

All patients fasted for at least 6 h before the 18F-FDG PET/CT study. 18F-FDG (370-555 MBq) was injected intravenously and scanning began 60 min later. No intravenous contrast agent was used. Two combined PET/CT in-line systems (Biograph DUO, Biograph Truepoint; Siemens Medical Solutions, Knoxville, TN, USA) were used to acquire all data. There were 6-8 bed positions, and the acquisition time was 2 min per bed position. CT began at the orbitomeatal line and progressed to the upper thigh (80 mA; 130 kVp; 5mm slice thickness). PET followed immediately over the same body region. The CT data were used for attenuation correction, and images were reconstructed using a standard ordered-subset expectation maximization algorithm. The axial spatial resolution was 6.5 mm and 4.5 mm at the center of the field of view. The time interval between preoperative PET/CT and curative surgery was 6.4 ± 5.7 days (mean ± SD, range 1-39 days).

All PET scans were reviewed and interpreted by two experienced nuclear medicine physicians (SJN & JHO) who were blinded to the clinical outcome and histopathologic findings. If the interpretations were different between the readers, the results were discussed until a consensus was reached. PET/CT was evaluated visually and quantitatively. In visual analysis, PET scan was considered as positive when perceptible FDG uptake that could be distinguished from physiologic gastric activity was noted at the site of the primary gastric tumor lesion as seen in the staging work-up endoscopy or enhanced CT. For quantitative analysis, one nuclear medicine physician (SJN) measured several metabolic parameters of primary tumor, according to the tumor defined by consensus while blinded to the patient outcome. SUV_max_ and SUV_peak_ of the primary gastric cancer were measured from all patients by drawing a volume of interest (VOI) at the primary tumor lesion. If no perceptible FDG uptake was noted at the tumor site, a fixed VOI was dropped at the site corresponding to the known gastric cancer, and the SUV_max_ and SUV_peak_ were measured by nuclear medicine physician. After SUV_mean_ of liver was measured from a 3 cm diameter spherical VOI dropped in the right side of the liver, TLR_max_ and TLR_peak_ were calculated. In addition, the MTV and TLG were computed in the patients with positive PET/CT scans. Threshold SUV of 3.0 was empirically selected and applied. All FDG PET parameters were measured by using the commercial software XD3 (Mirada Medical, Oxford, UK) [33, 39, 40].

### Follow up examinations and patient outcomes

**Figure 3:**
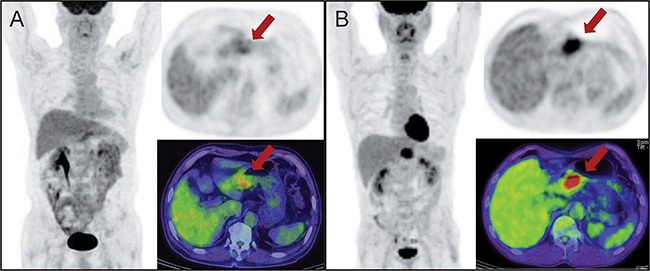
**A.** Preoperative 18F-PET/CT of a 64 year-old male shows mildly increased FDG uptake (SUV_max_ 3.6/TLR_max_ 2.0) in the gastric antrum. This patient was underwent subtotal gastrectomy and confirmed stage III gastric cancer (pT4aN3). The tumor was 6.0 × 5.5 cm sized, Bormann type II, poorly differentiated tubular adenocarcinoma. He followed up until 42 months without recurrence. **B.** A 55 year-old male, 18F-PET/CT for staging of gastric cancer presents localized intense hypermetabolic activity at the gastric antrum. The SUV_max_ and TLR_max_ of the tumor was 10.3 and 6.4, respectively. After subtotal gastrectomy, 4.5 × 3.0 cm sized, Bormann type III, poorly differentiated tubular adenocarcinoma was confirmed and the pathologic stage was pT4aN3. This patient had a recurrence 19 months after the surgery.

Patients underwent clinical follow up with serum biochemical tests, endoscopy and enhanced abdominopelvic CT every 3-6 months with or without follow up 18F-FDG PET/CT. When the clinical assessment, serum tumor markers, or imaging studies showed an abnormal finding, additional diagnostic studies or pathological confirmation were performed to check for cancer recurrence. Tumor recurrence was established by the combination of clinical follow up including tumor markers, findings on follow up CT scans, endoscopic features, and subsequent histopathologic diagnosis when indicated by endoscopic findings. OS was defined as the time from the curative resection to the time of death by any cause. RFS was defined as the time from the date of curative surgical resection to the time when recurrent tumor was first confirmed. Images from example 18F-FDG PET/CT cases are shown in Figure 3.

### Statistical analysis

Continuous variables are expressed as means (± standard deviation) or medians (range) and were compared using unpaired *t*-tests or ANOVA. The cut-off values for classifying the low and high metabolic FDG PET parameter groups for prognosis evaluation were determined using the maximal chi-square method of the R-system (version 2.13.0, http://www.R-project.org). The univariate and multivariate analyses with clinicopathologic factors were performed for assessing the association of RFS or OS and metabolic FDG PET parameters using Kaplan-Meier method with the log-rank test and the Cox proportional hazards model, respectively. The statistical analysis was performed using SAS software package (ver. 8.02, SAS Institute, Cary, NC, USA). All *p* values were two sided, and a *p* <0.05 was considered statistically significant.
